# Aromatase inhibitor-induced arthralgia ameliorated by Mediterranean diet and active lifestyle guided by continuous glucose monitoring: a case report and review of the literature

**DOI:** 10.3389/fonc.2024.1189287

**Published:** 2024-02-01

**Authors:** Kalin L. Wilson, Richard E. Grewelle, Tanya Gupta, Sun H. Kim, Tamiko R. Katsumoto

**Affiliations:** ^1^ Department of Medicine, Stanford University School of Medicine, Stanford, CA, United States; ^2^ Department of Biology, Stanford University, Stanford, CA, United States; ^3^ Department of Genetics, Stanford University School of Medicine, Stanford, CA, United States; ^4^ Department of Medicine, Division of Oncology, Stanford Cancer Institute, Stanford University School of Medicine, Stanford, CA, United States; ^5^ Department of Medicine, Division of Endocrinology, Stanford University School of Medicine, Stanford, CA, United States; ^6^ Department of Medicine, Division of Immunology and Rheumatology, Stanford University School of Medicine, Stanford, CA, United States

**Keywords:** hormone receptor-positive breast cancer, aromatase inhibitor-induced arthralgia, continuous glucose monitoring, Mediterranean diet, lifestyle medicine

## Abstract

Aromatase inhibitors (AIs) are a cornerstone adjuvant treatment of many hormone receptor-positive breast cancers, and nearly half of women taking aromatase inhibitors suffer from AI-induced arthralgia (AIA), also known as AI-associated musculoskeletal syndrome (AIMSS), for which there are limited evidence-based treatments. Pharmacologic management and complementary methods including supplements, exercise, physical therapy, yoga, acupuncture, and massage have all shown mixed results. Comprehensive diet and lifestyle strategies are understudied in AIA/AIMSS despite their disease-modifying effects across many chronic conditions. Here we report a case of a woman with stage 2 estrogen and progesterone receptor-positive invasive ductal carcinoma on adjuvant anastrozole whose AI-induced arthralgia was durably controlled through a Mediterranean plant-forward diet and daily physical activity guided by continuous glucose monitoring. We posit that diet and a lifestyle inclusive of daily physical activity constitute a low-cost, low-risk, and potentially high-reward strategy for controlling common AI-induced musculoskeletal symptoms and that more investigation in this arena, including well-designed randomized trials, is warranted.

## 1 Introduction

Breast cancer is the most common malignancy affecting 2.1 million women worldwide each year and second most common cause of cancer-related death among women in the United States (1, 2). Aromatase inhibitors (AIs) are central to the treatment of many estrogen and progesterone receptor-expressing breast cancers, which comprise 70-75% of all breast cancers (2, 3). However, they are associated with adverse effects, including a constellation of symptoms referred to as aromatase inhibitor-induced arthralgia (AIA) or more broadly as aromatase inhibitor-associated musculoskeletal syndrome (AIMSS), with criteria proposed by Niravath (4).

AIA/AIMSS classically presents with symmetrical joint pains affecting the hands, wrists, ankles, and/or knees; other symptoms include morning stiffness, myalgias, tenosynovitis, carpal tunnel syndrome, and trigger finger (4–6). This syndrome affects nearly 50% of women taking AIs (7) and contributes strongly to medication nonadherence and discontinuation (8). There are limited treatments for AIA/AIMSS other than drug discontinuation; pharmacologic and complementary management approaches have shown mixed results (9, 10). Furthermore, data on dietary interventions most often focus on a single diet modification or complementary diet supplement rather than on comprehensive diet change. Despite clear evidence that a healthy diet and active lifestyle can positively impact the course of many chronic conditions, there is a dearth of literature systematically investigating such interventions jointly for patients with AIA/AIMSS. Herein we present the case of a patient with AIA/AIMSS effectively controlled through comprehensive changes in diet and daily physical activity facilitated by continuous glucose monitor (CGM) use.

## 2 Case description

**Figure 1:**
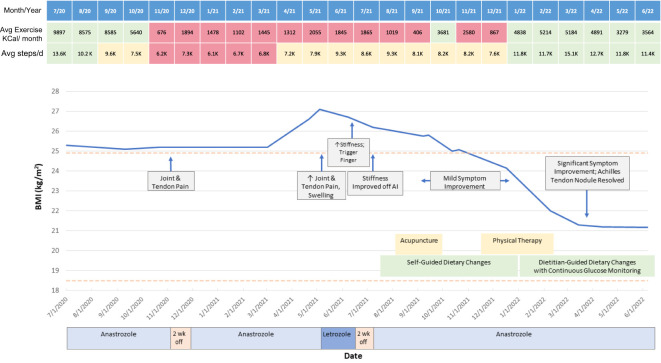
Timeline of patient symptoms, interventions, and BMI. Wk, week.

The patient, a 46-year-old female, presented with right breast mass in August 2017 and was diagnosed with stage 2, grade 3 estrogen receptor, progesterone receptor, and human epidermal growth factor receptor-expressing (ER+/PR+/HER2+) invasive ductal carcinoma (IDC) with associated ductal carcinoma *in situ* on MRI-guided biopsy. She underwent neoadjuvant therapy with 4 cycles of doxorubicin and cyclophosphamide (ddAC) and 16 cycles of paclitaxel, trastuzumab, and pertuzumab (THP), then proceeded to right partial mastectomy which revealed residual IDC, ER+/PR+/HER2+ (stage ypT1b(m)N0). Following a second resection due to proximity of IDC to the margin and a course of radiation therapy, she initiated tamoxifen in August 2018. She then transitioned to AI therapy with anastrozole in July 2020 when she was felt to be in menopause with multiple ultrasensitive estradiol levels<15 pg/mL (**Figure 1**).

Two months after initiating anastrozole, the patient developed ankle pain prompting medication discontinuation for two weeks. After restarting, she presented in February 2021 with bilateral hand and wrist pain and tingling, 30 minutes of morning joint stiffness, swelling, and decreased grip strength as well as right Achilles tendon pain and stiffness which she rated as an 8 out of 10 at worst. She preferred to remain on AI rather than returning to tamoxifen. Persistence of these symptoms prompted a switch from anastrozole to letrozole. However, on letrozole she experienced greater morning hand stiffness and new trigger finger such that letrozole was discontinued. Her stiffness then improved and she resumed anastrozole. She was referred to rheumatology for evaluation, where initial exam in July 2021 revealed right third and fourth PIP joint tenderness and a palpable, tender Achilles tendon nodule. Her labs were notable for normal ESR, borderline CRP, and negative ANA, RF, and CCP antibodies. She was diagnosed with AIA; alternative diagnoses considered included carpal tunnel syndrome and inflammatory arthritis including seronegative rheumatoid arthritis and the spondyloarthritides. Around this time, she was also diagnosed with prediabetes with a hemoglobin A1C (HgbA1c) of 6.3% and her pre-existing mild hepatic steatosis worsened to moderate nonalcoholic fatty liver disease based on ultrasound findings (see **Supplementary Figure 2**). She reported a diet with an abundance of high glycemic index foods such as bread, pasta, pizza, and several sweets (cookies, cakes). She thus opted for a trial of nonpharmacologic symptom management through modification of diet and physical activity. During this period she also briefly tried acupuncture without benefit and participated in three months of ankle physical therapy with some improvement in Achilles tendon pain but with persistence of hand stiffness and trigger finger symptoms.

**Figure 2:**
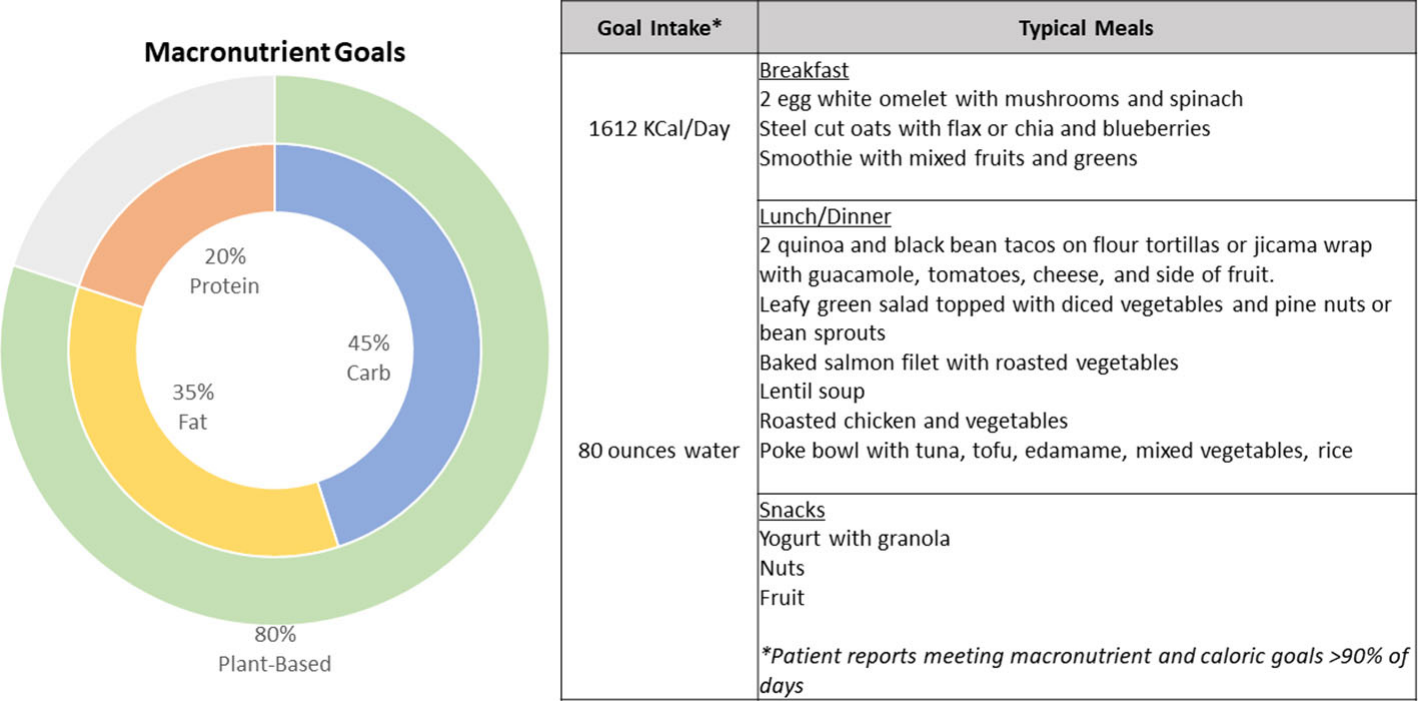
Summary characteristics of the patient’s dietitian-guided diet pattern with recommended macronutrient breakdown and examples of typical meals consumed by the patient.

**Figure 3:**
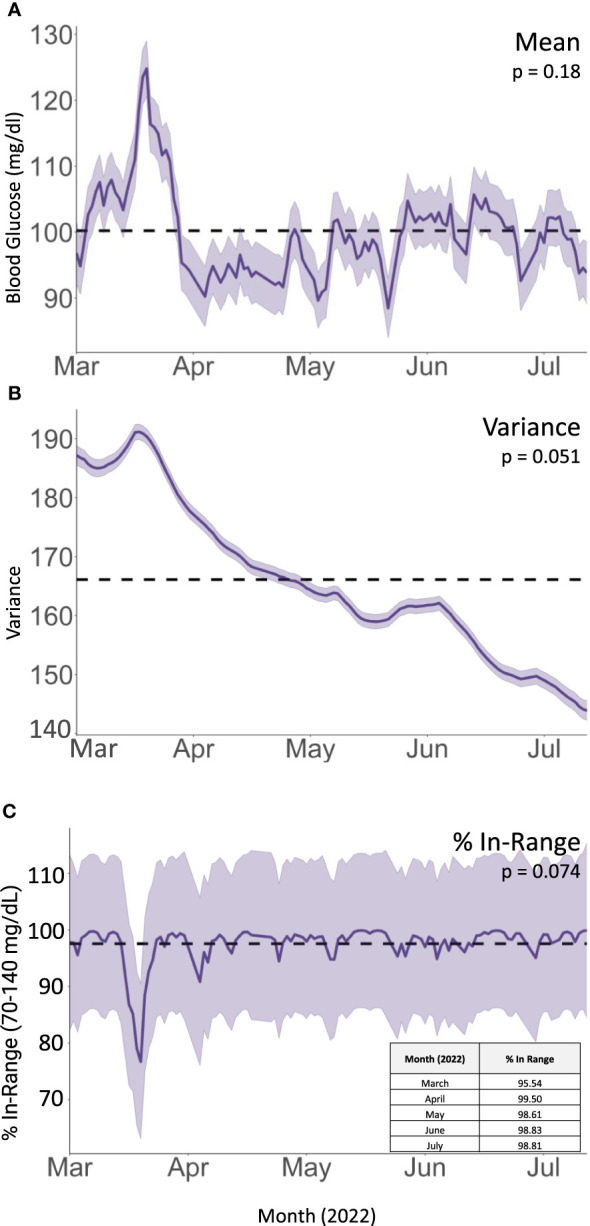
Integrated Nested Laplacian Approximation (INLA) model of **(A)** mean glucose values, **(B)** within-day variance in blood glucose values ( $\sigma^{2}$ ), and **(C)** fraction of CGM readings within target glucose range. Shaded area represents 95% confidence interval. Dotted line represents overall mean for the dataset.

On her follow up rheumatologic evaluation in March 2022, the patient had lost 17 pounds, a 12.5% loss from her peak body weight. She reported enrolling in a dietitian-supervised program most closely approximating a Mediterranean plant-forward diet with high intake of olive oil, fruits and vegetables, and less than 20% fish, poultry, or meat alternatives rather than red and/or processed meats and stated that she achieved her macronutrient goals (20% protein, 45% carbohydrates, 35% fat) over 90% of the time (**Figure 2**). Over the period of interest, she slowly transitioned more toward plant-based protein options. She also eliminated dairy, decreased the length of her daily food consumption window, and continued a consistent pattern of moderate aerobic exercise. She aimed to achieve approximately 40 minutes of exercise and 12,000 steps daily; however due to her significant arthralgias, her average daily step count and total number of calories burned (Peloton workout) initially declined from baseline (see **Figure 1**). Finally, she began using a CGM (Dexcom G6) to help lower daylong glucose and guide selection of lower glycemic index (GI) foods with less impact on postprandial glucose. The CGM also provided feedback regarding interventions to moderate rise in blood glucose of certain challenging foods. For example, she noted postprandial blood glucose elevations > 140 mg/dL after eating portions of cooked sweet potato, which led her to experiment with exercise and consumption techniques around this food item. She found that the same quantity of the same batch of cooked sweet potato would produce a less pronounced increase in blood glucose if she engaged in 20-30 minutes of moderate aerobic exercise (i.e., brisk walking or trampoline) before or after her meal or if she ate a portion of lean protein before consuming the sweet potato (**Supplementary Figure 1**). The patient incorporated these lessons into her daily practice, demonstrating that CGM usage can encourage exercise and diet modification. The CGM data reveal trends toward reduced glucose variability and increased time in the target range (70-140 mg/dL) (**Figure 3**). Consistent with these changes, her HgbA1C improved from 6.3 to 5.8% between December 2021 and August 2022. Her hepatic steatosis fully resolved by January 2023 (**Supplementary Figure 2**).

Subsequently, the patient’s stiffness, pain, and tingling significantly improved. Her Achilles tenosynovitis fully resolved and the nodule was no longer palpable, concordant with a substantial increase in her exercise patterns (daily step counts and calories burned in her Peloton workouts, **Figure 1**). She continues to tolerate AI therapy with anastrozole through the time of publication.

## 3 Conclusions

Herein we present the case of a 46-year-old female with ER+, PR+, HER2+ breast cancer on anastrozole who developed classic features meeting Niravath’s proposed criteria for AIA/AIMSS. After failing to improve with acupuncture, physical therapy, or switching AIs, she was able to durably control her symptoms non-pharmacologically through dietary changes, active lifestyle, and weight loss.

This patient’s course was notable in that after attempting several commonly cited management options, she was able to reduce her AIA/AIMSS symptoms through a diet most closely approximating a Mediterranean plant-forward diet coupled with other healthy lifestyle practices, an intervention without any adverse effects and indeed would be expected to positively impact health in other domains (e.g., type 2 diabetes, nonalcoholic fatty liver disease). To date, there have been no high-quality randomized controlled dietary intervention studies for the treatment of AIA/AIMSS. Importantly, a recruiting phase I/II trial plans to investigate an anti-inflammatory/Mediterranean diet for breast cancer patients on AIs (11).

Although some pharmacologic approaches have demonstrated success in curbing AIMSS symptoms, associated side effects may limit their tolerability. Although a large RCT testing duloxetine showed significant reduction in mean average pain score at week 12 in those with a BMI > 30 kg/m2, adverse effects were seen in 78% (vs. 50%) of participants on duloxetine compared with placebo (primarily fatigue, dry mouth, and headache) (12, 13). One week of low-dose prednisolone similarly reduced pain in about two thirds of individuals, with one third reporting persistent benefit at one month and one quarter at two months (14); while this might constitute an adequate temporizing measure, most patients continue AI therapy for years, and glucocorticoids would not be a viable therapeutic option over this period due to adverse metabolic effects and risk of bone loss.

Potentially lower-risk interventions include complementary approaches such as nutritional or herbal supplementation, acupuncture, meditation and mindfulness, and physical activity. It is challenging to assess the overall effectiveness of these interventions given the paucity and variable quality of data available in the literature. A recent Cochrane review on RCTs for AIA/AIMSS (15) identified 17 high-quality studies (4 prevention studies, 13 treatment studies) with over 2000 randomized patients, and the results are summarized in **Table 1**. Overall, there was very low-certainty evidence for the evaluated systemic therapies for the prevention or management of AIA/AIMSS. Single-agent dietary supplements such as omega-3 fatty acids and Vitamin D supplementation have tended not to induce a durable reduction in pain (**Table 1**). Quality of the studies included in the Cochrane review was variable, with differences in endpoints, timing of measurements, study conduct, and risk of bias. Therapies evaluated in this systematic review included etoricoxib (17), testosterone (18, 19), duloxetine (12), calcitonin (20), omega-3 fatty acid supplementation (21, 22), vitamin D3 supplementation (23–26), tart cherry (27), bionic tiger bone capsules (28), Yi Shen Jian Gu granules (29), emu oil (30), and Cat’s claw (31). Standardization of the measurement of outcomes in AIA/AIMSS, including patient reported outcomes (PROs), and standardization of the time points for assessment would improve research quality and reduce heterogeneity in comparing studies (15).

**Table 1:** Randomized controlled trials included in AIA/AIMSS Cochrane Reviews (15, 16).

	Author, year	Trial Type	N	Study details	Outcome	Caveats
MEDICATIONS						
Duloxetine	Henry 2018	Treatment	299	Significant reduction in mean average pain score (BPI-SF) at wk 12 in BMI >30 kg/m2 but not in BMI< 30 kg/m2	Positive	AEs: 78% duloxetine, 50% PBO (P<0.001), most common AEs fatigue, dry mouth, headache
Etoricoxib	Rosati 2011 (abstract only)	Treatment	182	2o EP: MSK pain significantly lower in treatment arm (50/73) than control (16/67) [RR: 2.1, 95%CI (1.29-3.43), P=0.002]	Positive	Abstract only, high dropout rate
Testosterone	Birrell 2009 (abstract only)	Treatment	90	Decreased pain VAS scores inof 43% testosterone 40 mg, 70% testosterone 80 mg vs 35% PBO, P=0.06 and P=0.04 vs PBO respectively	Positive	Abstract only, supported by Astra Zeneca
	Cathcart-Rake 2020	Treatment	227	No difference in pain or stiffness at 3 and 6 mos (BPI-AIA, item #3)	Negative	
Calcitonin	Liu 2014	Treatment	91	Pain VAS significantly decreased -3 (Calcitonin 200 IU group) vs -1 (PBO), P<0.01	Positive	Baseline difference in VAS (5.38 Calcitonin vs 4.48 Control, P=0.006), 1o EP not specified
SUPPLEMENTS						
Vitamin D3	Khan 2017	Prevention	160	Prevention of protocol defined AIMSS event 37% in high dose vitamin D 30,000 IU/week vs 51% PBO (P=0.069)	Negative	
	Niravath 2019	Prevention	93	AIMSS developed in 54% high dose vit D 50,000 IU/wk vs 57% standard dose vit D 2000 IU daily	Negative	
	Rastelli 2011	Treatment	60	FIQ pain (3.3 vs 4.6, P=0.0045), BPI worst pain (3.6 vs 5.1, P=0.04), BPI avg pain (2.7 vs 3.7, P=0.0067) better in high dose vitamin D vs PBO at 2 mos	Positive	
	Shapiro 2016	Treatment	116	No significant differences in BCPT-MS, WOMAC, AUSCAN, PROMIS, Hand grip	Negative	
Omega 3 fatty acids	Lustberg 2018	Prevention	44	2o EP: Mean BPI-SF score did not change significantly by time or treatment arm	Negative	
	Hershman 2015	Treatment	262	Mean BPI-SF score decreased by 1.74 vs 1.49 (wk 12) and 2.22 vs 1.81 (wk 24) (P=0.58)	Negative	
Tart Cherry	Shenouda 2022	Treatment	48	Mean pain (VAS) decrease by 34.7% tart cherry vs 1.4% placebo at wk 6 (P=0.034)	Positive	12 pts excluded from analysis
Yi Shen Jian Gu granules	Peng 2018	Treatment	84	Worst pain scores (BPI-SF) decreased by 3.10 pts (50.2%) YSJG vs. 1.63 pts (26.9%) PBO, P=0.001	Positive	Limited generalizability (study population all Asian), clear diagnostic criteria and specific measures for AIMSS absent
Bionic tiger bone capsules	Li 2017	Treatment	70	New or worsening joint symptoms in 22.9% tiger bone vs. 60% PBO wk 12 (P<0.001)	Positive	
Blue Citrus	Massimino 2011 (abstract only)	Treatment	37	Mean VAS score 2.98 Blue Citrus vs 3.92 PBO (P=0.0203) at 30 d but by end of study VAS scores were 2.6 vs 3.0 (180 d)	?	Abstract only, limited data available
Emu Oil	Chan 2017	Treatment	87	No statistically significant benefit in joint pain at week 8 (VAS)	Negative	
Cat’s claw	Sordi 2019	Treatment	70	Uncaria tomentosa was not more effective than placebo (BPI, DASH, VAS pain)	Negative	
EXERCISE						
Supervised mixed aerobic/resistance training, vs usual care	Irwin 2015	Treatment	121	Worst joint pain scores decreased by 1.6 pts (29%) vs 0.2 pt increases (3%) at 12 months (P<0.001)	Positive	
Unsupervised walking, vs waiting list control	Nyrop 2017	Treatment	62	Worst pain (WOMAC) not statistically significantly different between intervention and control	Negative	Improvements in WOMAC stiffness, difficulty and total score
Patient’s choice of 3 exercise intensity levels, vs usual care -	Tamaki 2018	Treatment	102	Trends for pain interference at 12 months did not reach statistical significance	Negative	
Supervised followed by independent Nordic Walking, vs usual care	Fields 2016	Treatment	159	BPI-SF decreased -1.5 (intervention group) vs. -2.5 (control group)	Negative	Feasibility study
Supervised followed by unsupervised mixed aerobic/resistance training	Lohrisch 2011 (abstract only)	Treatment	22	Only 20 evaluable subjects, mean SF36 improved in 6 (55%) and 7 (64%)	Negative	Abstract only, limited data available
Supervised mixed aerobic/resistance training, vs usual care	Sanmugarajah 2017 (abstract only)	Prevention	20	Mean pain scores (BPI) increased by 1 unit (exercise group) vs. 5 units (PBO) at 12 mos (P>0.05)	Negative	Abstract only, limited data available
Supervised exercise program vs unsupervised walking	Varadarajan 2016	Treatment	27	Significant improvement in grip strength	Positive	Abstract only, limited data available

AE, adverse events; AUSCAN, Australian/Canadian osteoarthritis hand index version 3.1; BCPT-MS, breast cancer prevention trial-musculoskeletal scale; BPI-AIA, brief pain inventory-aromatase induced arthralgia; BPI-SF, brief pain inventory-short form; DASH, disability Arm, Shoulder, and Hand; FIQ, fibromyalgia impact questionnaire; PBO, placebo; VAS, visual analog scale; WOMAC, Western Ontario and McMaster osteoarthritis index version 3.1; 1o EP, primary endpoint; 2o EP, secondary endpoint.

Another recent Cochrane review evaluated exercise as a treatment for AIA/AIMSS and included 7 studies (1 prevention study, 6 intervention studies) with 400 randomized participants (see **Table 1**) (32). Considerable heterogeneity was noted amongst the trials, and the meta-analysis provided no clear evidence that exercise was beneficial in AIMSS. Other meta-analyses have revealed trends toward improvement in pain scores with physical exercise and acupuncture but no significant signal for mindfulness and relaxation techniques (9, 10, 33). Briefly, diverse exercise interventions were represented, with the best signal originating from trials of mixed aerobic/resistance programs, while walking interventions and tai chi were less successful. One study by Irwin et al. (2015 JCO) showed significant improvement in worst joint pain scores in patients randomized to the exercise arm, consisting of at least 150 minutes per week of aerobic exercise and supervised strength training twice per week. Studies on yoga were too sparse and heterogeneous to allow for a systematic assessment, but generally showed improvement without serious adverse effects. Acupuncture provided pain relief in several available studies, with the substantial caveat that sham acupuncture often provided a similar benefit.

Due to its observational nature, this case study has inherent limitations. A key limitation is that the mechanism for AIA improvement is unclear as multiple changes were made simultaneously. Nevertheless, the combination of changes led to our patient decreasing BMI from a peak of around 27 to 21 over the period of interest, which can have many metabolic benefits that may have mediated her improvement in AIA. However, caution is advised in the application of these results to individuals with normal BMI. BMI > 30 or weight > 80 kg was associated with increased risk of developing joint symptoms in the ATAC (Arimidex Tamoxifen Alone or in Combination) and IES (Intergroup Exemestane Study) cohorts, respectively (34, 35). However, other studies have demonstrated that BMI did not predict time to AI discontinuation due to treatment-related symptoms, suggesting perhaps that obesity predisposes to AIMSS symptoms but does not reliably predict their severity. Further complicating matters, although obesity positively correlates with onset of AIA/AIMSS, a cross-sectional survey found that overweight women (BMI 25 to 30) experienced joint symptoms less frequently than their counterparts with BMI< 25 or > 30. Estrogen signaling is known to modulate glucose and lipid metabolism and immune function; a healthy, antioxidant-rich diet may therefore counteract AI-induced changes by enhancing insulin sensitivity, decreasing body fat, and reducing inflammation with or without augmentation by weight loss. In this patient with co-existing metabolic abnormalities (prediabetes and NAFLD) suggesting insulin resistance, weight loss likely was a contributor to her symptomatic improvement. Although it is difficult to parse the individual roles diet, exercise and weight loss as mediators given their interrelatedness, future RCTs could stratify patients based on BMI to better address this issue.

In comparison to patients enrolled in the exercise RCT for AIA (Irwin et al., 2015 JCO), our patient was younger at age 46, compared with the average age of 62 in the exercise group and 60.5 in the usual care group. Her peak BMI of 27 was slightly lower than the average BMI of 30 and 28.7 (exercise and usual care groups, respectively) and her degree of weight loss was greater (-12.5% compared with -2.4% in the exercise group and 0% in the usual care group). She was considerably more active at baseline, with approximately 525 minutes of physical activity a week (assuming 6000 steps a day), which was substantially greater than 54.8 and 60.7 minutes per week in the exercise and usual care groups, respectively. Thus, in designing future RCTs, we speculate that more ambitious exercise targets could yield more dramatic results.

This patient’s favorable outcome suggests that diet coupled with a pattern of daily physical activity can be a promising, cost-effective and low-risk intervention for many patients suffering from AIA/AIMSS. This is in line with existing evidence suggesting beneficial effects of Mediterranean and plant-based diets on pain control in inflammatory arthritis, including rheumatoid arthritis (36), and with their known disease-modifying activity in conditions mediated by chronic low-grade inflammatory states, including atherosclerotic cardiovascular disease and cancer (32, 37). It also highlights the power of technologies like CGM, which was instrumental in empowering this patient to make impactful changes. A potential RCT could leverage meal delivery services that adhered to a Mediterranean plant-forward dietary pattern consisting of the macronutrient proportions described in **Figure 2**. In addition, group classes with a dietitian could be incorporated into the intervention to ensure optimal interpretation of CGM data as well as dietary suggestions on how to minimize the glycemic impact of foods. Additional studies are required to pinpoint key beneficial diet and exercise practices for AIA/AIMSS and the mechanisms thereof.

In summary, we propose that thoughtfully designed studies testing the use of a Mediterranean plant-forward diet accompanied by regular exercise should be pursued. Performing randomized controlled trials (RCTs) to assess multimodal interventions is challenging but feasible (38), and the use of mobile technologies (e.g. CGMs, step counters, etc.) to quantitatively assess adherence to dietary and exercise interventions can improve the fidelity of multimodal lifestyle intervention RCTs. AIA/AIMSS is a condition that impairs quality of life and interrupts a potentially life-saving therapy for substantial numbers of patients with breast cancer, and there is a clear need for more effective evidence-based AIA/AIMSS treatment strategies.

## Patient perspective

I developed life impacting side effects from taking an aromatase inhibitor (AI). I dropped things because of tingling hands, slept with wrist braces because of carpal tunnel-like pain, took extra time to get out of bed due to stiff joints, and had difficulty walking with a large bump on my Achilles tendon. I was in pain, couldn’t easily exercise, gained weight, developed a Non-Alcoholic Fatty Liver, showed elevated cholesterol levels, and my A1C was just under the range for Type 2 Diabetes.

While waiting to start a new medication to relieve the AI side effects, I addressed my weight and other health issues. I found a team of registered dieticians who introduced me to the Mediterranean Diet, calculated my specific macronutrient goals and supervised me while I wore a continuous glucose monitor (CGM). The Mediterranean Diet told me what to eat. The CGM data helped me understand when and how much to eat, which foods (including some unexpected ones) trigger blood glucose spikes for me, and when to get up and move my body. Together, all this information helped me keep my blood glucose steady.

I lost a significant amount of weight and many of my AI side effects disappeared. Now, my hands no longer tingle. The bump on my Achilles tendon went away and I can walk hills again. I don’t need wrist braces to sleep. I have maintained my weight loss. Both my A1C and cholesterol levels have dropped. My latest abdominal ultrasound presented my liver appearance as normal - my fatty liver is gone. My AI lowers my risk for breast cancer recurrence. I am thrilled I can better tolerate this medication and keep taking it for the planned amount of time. I am excited I did this by simply making dietary and lifestyle changes and without having to add a new medication.

## Data availability statement

The raw data supporting the conclusions of this article will be made available by the authors, without undue reservation.

## Ethics statement

Written informed consent was obtained from the individual(s) for the publication of any potentially identifiable images or data included in this article.

## Author contributions

KW and TK conducted patient interviews to gather information regarding symptoms and dietary practices. KW abstracted data from the patient chart and drafted the initial manuscript. RG performed the statistical analysis of CGM data. TK, SH, and TG provided meaningful review and revision of the intellectual content of multiple drafts. All authors approved the submitted version.

## Supplementary material

Supplementary Figure 1 A representative example of CGM-guided changes to diet and intake and its impacts on postprandial blood glucose trends. Blue trendline (3/15/2022): Consumption of one serving of Japanese sweet potato alone without aerobic activity produced a blood glucose spike > 140 mg/dl between 1 and 2 hours after eating. Orange trendline (6/20/2022): Consumption of one serving of the same batch of frozen and reheated Japanese sweet potato topped with quinoa, black beans, avocado, and tomatoes with a side of corn, followed by a 30 minute brisk walk. Gray trendline (6/27/2022): Consumption of one serving of the same sweet potato with ground turkey, grilled and blanched vegetables followed by a 30 minute brisk walk.

Supplementary Figure 2 Overlay of serial ultrasound measurements of hepatic steatosis on timeline reveals a delayed resolution of hepatic steatosis following diet and lifestyle modifications.

## References

[1] Islami F Ward EM Sung H Cronin KA Tangka FKL Sherman RL . Annual report to the nation on the status of cancer, part 1: national cancer statistics. J Natl Cancer Inst (2021) 113(12):1648–69. doi: 10.1093/jnci/djab131 PMC8634503 34240195

[2] Boszkiewicz K Piwowar A Petryszyn P . Aromatase inhibitors and risk of metabolic and cardiovascular adverse effects in breast cancer patients-A systematic review and meta-analysis. J Clin Med (2022) 11(11):3133. doi: 10.3390/jcm11113133 35683517 PMC9181297

[3] Harbeck N Penault-Llorca F Cortes J Gnant M Houssami N Poortmans P . Breast cancer. Nat Rev Dis Primers (2019) 5(1):66. doi: 10.1038/s41572-019-0111-2 31548545

[4] Niravath P . Aromatase inhibitor-induced arthralgia: a review. Ann Oncol (2013) 24(6):1443–9. doi: 10.1093/annonc/mdt037 23471104

[5] Morales L Pans S Paridaens R Westhovens R Timmerman D Verhaeghe J . Debilitating musculoskeletal pain and stiffness with letrozole and exemestane: associated tenosynovial changes on magnetic resonance imaging. Breast Cancer Res Treat (2007) 104(1):87–91. doi: 10.1007/s10549-006-9394-6 17061044

[6] Sestak I Sapunar F Cuzick J . Aromatase inhibitor-induced carpal tunnel syndrome: results from the ATAC trial. J Clin Oncol (2009) 27(30):4961–5. doi: 10.1200/JCO.2009.22.0236 19752338

[7] Beckwée D Leysen L Meuwis K Adriaenssens N . Prevalence of aromatase inhibitor-induced arthralgia in breast cancer: a systematic review and meta-analysis. Support Care Cancer (2017) 25(5):1673–86. doi: 10.1007/s00520-017-3613-z 28204994

[8] Henry NL Giles JT Ang D Mohan M Dadabhoy D Robarge J . Prospective characterization of musculoskeletal symptoms in early stage breast cancer patients treated with aromatase inhibitors. Breast Cancer Res Treat (2008) 111(2):365–72. doi: 10.1007/s10549-007-9774-6 PMC3081690 17922185

[9] Roberts K Rickett K Greer R Woodward N . Management of aromatase inhibitor induced musculoskeletal symptoms in postmenopausal early Breast cancer: A systematic review and meta-analysis. Crit Rev Oncol Hematol (2017) 111:66–80. doi: 10.1016/j.critrevonc.2017.01.010 28259297

[10] Yang GS Kim HJ Griffith KA Zhu S Dorsey SG Renn CL . Interventions for the treatment of aromatase inhibitor-associated arthralgia in breast cancer survivors: A systematic review and meta-analysis. Cancer Nurs (2017) 40(4):E26–41. doi: 10.1097/NCC.0000000000000409 27333128

[11] Carpenter CL . Dietary and Exercise Interventions in Reducing Side Effects in Patients With Stage I-IIIa Breast Cancer Receiving Aromatase Inhibitors. ClinicalTrials.gov. Available at: https://clinicaltrials.gov/ct2/show/NCT03953157.

[12] Henry NL Unger JM Schott AF Fehrenbacher L Flynn PJ Prow DM . Randomized, multicenter, placebo-controlled clinical trial of duloxetine versus placebo for aromatase inhibitor-associated arthralgias in early-stage breast cancer: SWOG S1202. J Clin Oncol (2018) 36(4):326–32. doi: 10.1200/JCO.2017.74.6651 PMC5805479 29136387

[13] Henry NL Unger JM Till C Schott AF Crew KD Lew DL . Association between body mass index and response to duloxetine for aromatase inhibitor-associated musculoskeletal symptoms in SWOG S1202. Cancer (2019) 125(12):2123–9. doi: 10.1002/cncr.32024 PMC6675412 30861098

[14] Kubo M Onishi H Kuroki S Okido M Shimada K Yokohata K . Short-term and low-dose prednisolone administration reduces aromatase inhibitor-induced arthralgia in patients with breast cancer. Anticancer Res (2012) 32(6):2331–6. 22641670

[15] Roberts KE Rickett K Chatfield MD Woodward NE . Systemic therapies for preventing or treating aromatase inhibitor-induced musculoskeletal symptoms in early breast cancer. Cochrane Database Syst Rev (2018) (1):CD013167. doi: 10.1002/14651858.CD013167 PMC8743877 35005781

[16] Roberts KE Rickett K Feng S Vagenas D Woodward NE . Exercise therapies for preventing or treating aromatase inhibitor-induced musculoskeletal symptoms in early breast cancer. Cochrane Database Syst Rev (2020) 1(1):CD012988. doi: 10.1002/14651858.CD012988.pub2 31994181 PMC6987034

[17] Rosati MS Di Seri M Baciarello G Lo Russo V Grassi P Marchetti L . Etoricoxib and anastrozole in adjuvant early breast cancer: ETAN trial (phase III). J Clin Oncol (2011) 29(15_suppl):533. doi: 10.1200/jco.2011.29.15_suppl.533

[18] Birrell S Tilley W . Testosterone undecanoate treatment reduces joint morbidities induced by anastrozole therapy in postmenopausal women with breast cancer: results of a double-blind, randomized phase II trial. Cancer Res (2009) 69(24_Supplement):804–4. doi: 10.1158/0008-5472.SABCS-09-804

[19] Cathcart-Rake E Novotny P Leon-Ferre R Le-Rademacher J Storrick EM Adjei AA . A randomized, double-blind, placebo-controlled trial of testosterone for treatment of postmenopausal women with aromatase inhibitor-induced arthralgias: Alliance study A221102. Support Care Cancer (2021) 29(1):387–96. doi: 10.1007/s00520-020-05473-2 PMC7644633 32372176

[20] Liu P Yang DQ Xie F Zhou B Liu M . Effect of calcitonin on anastrozole-induced bone pain during aromatase inhibitor therapy for breast cancer. Genet Mol Res (2014) 13(3):5285–91. doi: 10.4238/2014.July.24.7 25078584

[21] Lustberg MB Orchard TS Reinbolt R Andridge R Pan X Belury M . Randomized placebo-controlled pilot trial of omega 3 fatty acids for prevention of aromatase inhibitor-induced musculoskeletal pain. Breast Cancer Res Treat (2018) 167(3):709–18. doi: 10.1007/s10549-017-4559-z PMC5809189 29101597

[22] Hershman DL Unger JM Crew KD Awad D Dakhil SR Gralow J . Randomized multicenter placebo-controlled trial of omega-3 fatty acids for the control of aromatase inhibitor-induced musculoskeletal pain: SWOG S0927. J Clin Oncol (2015) 33(17):1910–7. doi: 10.1200/JCO.2014.59.5595 PMC4451174 25940724

[23] Khan QJ Kimler BF Reddy PS Sharma P Klemp JR Nydegger JL . Randomized trial of vitamin D3 to prevent worsening of musculoskeletal symptoms in women with breast cancer receiving adjuvant letrozole. The VITAL trial. Breast Cancer Res Treat (2017) 166(2):491–500. doi: 10.1007/s10549-017-4429-8 28770449

[24] Niravath P Hilsenbeck SG Wang T Jiralerspong S Nangia J Pavlick A . Randomized controlled trial of high-dose versus standard-dose vitamin D3 for prevention of aromatase inhibitor-induced arthralgia. Breast Cancer Res Treat (2019) 177(2):427–35. doi: 10.1007/s10549-019-05319-4 31218477

[25] Rastelli AL Taylor ME Gao F Armamento-Villareal R Jamalabadi-Majidi S Napoli N . Vitamin D and aromatase inhibitor-induced musculoskeletal symptoms (AIMSS): a phase II, double-blind, placebo-controlled, randomized trial. Breast Cancer Res Treat (2011) 129(1):107–16. doi: 10.1007/s10549-011-1644-6 21691817

[26] Shapiro AC Adlis SA Robien K Kirstein MN Liang S Richter SA . Randomized, blinded trial of vitamin D3 for treating aromatase inhibitor-associated musculoskeletal symptoms (AIMSS). Breast Cancer Res Treat (2016) 155(3):501–12. doi: 10.1007/s10549-016-3710-6 PMC5260816 26868123

[27] Shenouda M Copley R Pacioles T Lebowicz Y Jamil M Akpanudo S . Effect of tart cherry on aromatase inhibitor-induced arthralgia (AIA) in nonmetastatic hormone-positive breast cancer patients: A randomized double-blind placebo-controlled trial. Clin Breast Cancer (2022) 22(1):e30–6. doi: 10.1016/j.clbc.2021.06.007 34275765

[28] Li Y Zhang Z Cui F Liu J Wang Y Jiang J . Traditional chinese medicine bionic tiger bone powder for the treatment of AI-associated musculoskeletal symptoms. Evid Based Complement Alternat Med (2017) 2017:2478565. doi: 10.1155/2017/2478565 28250792 PMC5307008

[29] Peng N Yu M Yang G Fu Q Xu Y Yu J . Effects of the Chinese medicine Yi Shen Jian Gu granules on aromatase inhibitor-associated musculoskeletal symptoms: A randomized, controlled clinical trial. Breast (2018) 37:18–27. doi: 10.1016/j.breast.2017.08.003 29059538

[30] Chan A De Boer R Gan A Willsher P Martin R Zissiadis Y . Randomized phase II placebo-controlled study to evaluate the efficacy of topical pure emu oil for joint pain related to adjuvant aromatase inhibitor use in postmenopausal women with early breast cancer: JUST (Joints Under Study). Support Care Cancer (2017) 25(12):3785–91. doi: 10.1007/s00520-017-3810-9 28691132

[31] Sordi R Nastri Castro S Thaumaturgo Lera A Nonato Irene M de Melo Farinazzo M Sette C . Randomized, double-blind, placebo-controlled phase II clinical trial on the use of uncaria tomentosa (Cat’s claw) for aromatase inhibitor-induced arthralgia: A pilot study. J Natural Remedies (2019) 19(1):24–31. doi: 10.18311/jnr/2019/22867

[32] Morze J Danielewicz A Przybyłowicz K Zeng H Hoffmann G Schwingshackl L . An updated systematic review and meta-analysis on adherence to mediterranean diet and risk of cancer. Eur J Nutr (2021) 60(3):1561–86. doi: 10.1007/s00394-020-02346-6 PMC7987633 32770356

[33] Bae K Lamoury G Carroll S Morgia M Lim S Baron-Hay S . Comparison of the clinical effectiveness of treatments for aromatase inhibitor-induced arthralgia in breast cancer patients: A systematic review with network meta-analysis. Crit Rev Oncol Hematol (2023) 181:103898. doi: 10.1016/j.critrevonc.2022.103898 36535489

[34] Sestak I Cuzick J Sapunar F Eastell R Forbes JF Bianco AR . Risk factors for joint symptoms in patients enrolled in the ATAC trial: a retrospective, exploratory analysis. Lancet Oncol (2008) 9(9):866–72. doi: 10.1016/S1470-2045(08)70182-7 18703382

[35] Mieog JSD Morden JP Bliss JM Coombes RC van de Velde CJH . IES Steering Committee. Carpal tunnel syndrome and musculoskeletal symptoms in postmenopausal women with early breast cancer treated with exemestane or tamoxifen after 2-3 years of tamoxifen: a retrospective analysis of the Intergroup Exemestane Study. Lancet Oncol (2012) 13(4):420–32. doi: 10.1016/S1470-2045(11)70328-X 22265698

[36] Schönenberger KA Schüpfer A-C Gloy VL Hasler P Stanga Z Kaegi-Braun N . Effect of anti-inflammatory diets on pain in rheumatoid arthritis: A systematic review and meta-analysis. Nutrients (2021) 13(12):4221. doi: 10.3390/nu13124221 34959772 PMC8706441

[37] Widmer RJ Flammer AJ Lerman LO Lerman A . The Mediterranean diet, its components, and cardiovascular disease. Am J Med (2015) 128(3):229–38. doi: 10.1016/j.amjmed.2014.10.014 PMC4339461 25447615

[38] Younge JO Kouwenhoven-Pasmooij TA Freak-Poli R Roos-Hesselink JW Hunink MM . Randomized study designs for lifestyle interventions: a tutorial. Int J Epidemiol (2015) 44(6):2006–19. doi: 10.1093/ije/dyv183 26377509

