# Supplementary figures and images for: Aromatase inhibitor-induced arthralgia ameliorated by Mediterranean diet and active lifestyle guided by continuous glucose monitoring: a case report and review of the literature
Source: Front Oncol. 2024 Feb 1;14:1189287. doi: 10.3389/fonc.2024.1189287 (PMC10867103; doi:10.3389/fonc.2024.1189287)

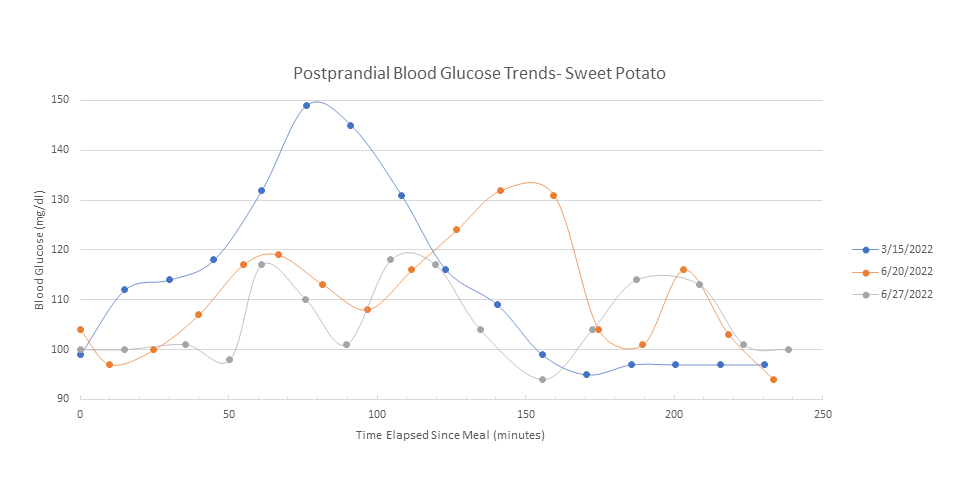

Supplement: Supplementary Figure 1 — A representative example of CGM-guided changes to diet and intake and its impacts on postprandial blood glucose trends. Blue trendline (3/15/2022): Consumption of one serving of Japanese sweet potato alone without aerobic activity produced a blood glucose spike > 140 mg/dl between 1 and 2 hours after eating. Orange trendline (6/20/2022): Consumption of one serving of the same batch of frozen and reheated Japanese sweet potato topped with quinoa, black beans, avocado, and tomatoes with a side of corn, followed by a 30 minute brisk walk. Gray trendline (6/27/2022): Consumption of one serving of the same sweet potato with ground turkey, grilled and blanched vegetables followed by a 30 minute brisk walk. [file Image_1.tif]

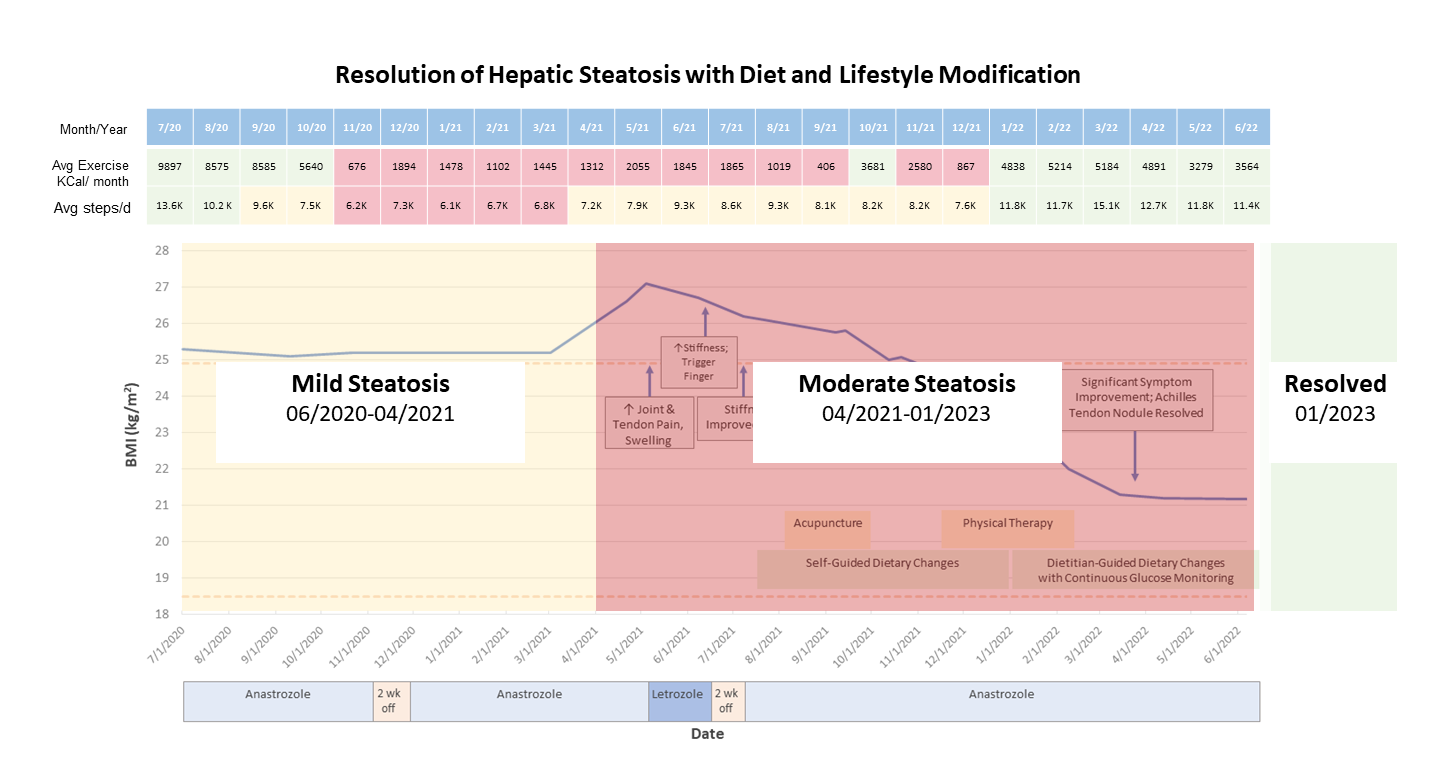

Supplement: Supplementary Figure 2 — Overlay of serial ultrasound measurements of hepatic steatosis on timeline reveals a delayed resolution of hepatic steatosis following diet and lifestyle modifications. [file Image_2.tif]
